# Pneumocéphalie compliquant un Blood patch réalisé pour des céphalées post césarienne

**DOI:** 10.11604/pamj.2014.19.201.5614

**Published:** 2014-10-24

**Authors:** Nezha Oudghiri, Mouhssine Doumiri

**Affiliations:** 1 Département d'Anesthésie Réanimation Obstétricale, Hôpital Maternité Souissi, Université Mohamed V, Rabat, Maroc

**Keywords:** pneumocephalie, Blood patch, césarienne, TDM, pneumocephalus, Blood patch, caesarean section, TDM

## Image en medicine

Patiente âgée de 24 ans, consulte aux urgences obstétricales pour des céphalées et des vomissements apparus 48 heures après une césarienne urgente faitesous rachi anesthésie. Ces céphalées étaient intenses, positionnelles, et rebelles au traitement médical pris pendant sept jours (réhydratation, repos, caféine, paracétamol). L'examen clinique n'a pas montré de déficit sensitivomoteur ou de troubles neurosensoriels, la nuque était souple, la température était de 37,2. L'examen cardiorespiratoire était sans particularités. Devant ces céphalées persistantes et invalidantes, le diagnostic d'une brèche dure mérienne a été posé et on a décidé de faire un Blood Patch. Le niveau choisi était L4-L5, en dessous de la ponction précédente avec repérage grâce à une seringue remplie d'air et l'injection de 15 ml de sang autologue prélevé au niveau du pli du coude, ce volume a été déterminé à partir d'une sensation d'une tension au niveau lombaire rapportée par la patiente. Lors de la procédure il y a eu une effraction de la dure mère avec issue du liquide céphalorachidien. Au troisième jour et devant la persistance et surtout l'aggravation des céphalées, une TDM cérébrale a été demandée et a objectivé lapneumocéphalie. L’évolution a été marquée par le tarissement spontané et complet des douleurs après 24heures d'hospitalisation en réanimationaprès laquelle la patiente est sortie à domicile. Cette observation souligne l'importance de réaliser un scanner cérébral devant une non résolution par un Blood patch des céphalées post brèche dure mérienne.

**Figure 1:**
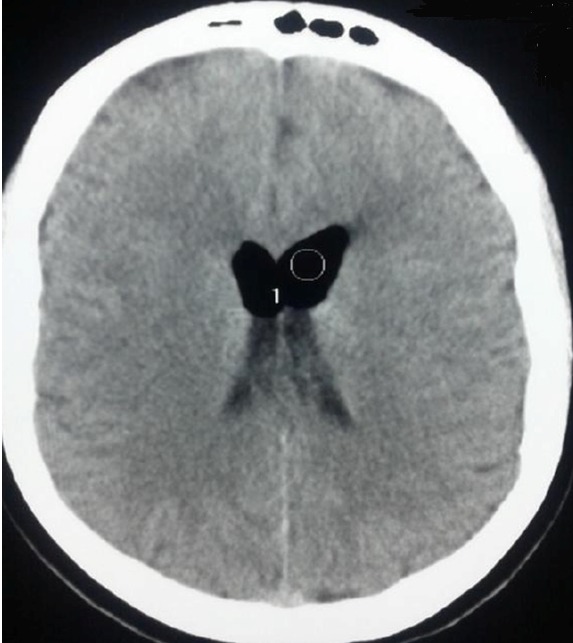
Présence d'air au niveau des citernes et des ventricules latéraux

